# Individual differences in emoji comprehension: Gender, age, and culture

**DOI:** 10.1371/journal.pone.0297379

**Published:** 2024-02-14

**Authors:** Yihua Chen, Xingchen Yang, Hannah Howman, Ruth Filik

**Affiliations:** School of Psychology, University of Nottingham, University Park, Nottingham, United Kingdom; Universita Telematica Pegaso, ITALY

## Abstract

Emoji are an important substitute for non-verbal cues (such as facial expressions) in online written communication. So far, however, little is known about individual differences regarding how they are perceived. In the current study, we examined the influence of gender, age, and culture on emoji comprehension. Specifically, a sample of 523 participants across the UK and China completed an emoji classification task. In this task, they were presented with a series of emoji, each representing one of six facial emotional expressions, across four commonly used platforms (Apple, Android, WeChat, and Windows). Their task was to choose from one of six labels (happy, sad, angry, surprised, fearful, disgusted) which emotion was represented by each emoji. Results showed that all factors (age, gender, and culture) had a significant impact on how emojis were classified by participants. This has important implications when considering emoji use, for example, conversation with partners from different cultures.

## Introduction

Communication plays an essential role in our daily lives, during which we convey information to others using both verbal (e.g., speech) and non-verbal (e.g., facial expression) behaviour [1] With the development of Information and Communication Technologies, computer-mediated communication (CMC) is widespread, and growing exponentially, especially text-based communication [2]. There are many advantages of CMC, including facilitating communication regardless of time and location, facilitating the archiving of information, and maintaining the continuity of relationships [3]. Although online communication is convenient and efficient, Kaye et al. [1] found that many people would choose to communicate face-to-face when they would like to convey emotions, since non-verbal information would be lost in the process of online communication and may lead to unnecessary misunderstanding. Emoticons and emoji were introduced as a compensatory mechanism [4]. Given the increasing importance of emoji in everyday communication, the present study aims to investigate whether there are individual differences in the emotional labels that people choose to assign to emoji representing facial emotional expressions (happy, sad, angry, fearful, surprise, disgusted). The specific individual differences examined in the current study are gender, age, and culture (UK and China).

### Emoticons and emoji

Emoticons, such as:), are composed of keyboard characters, and were first designed by Scott Fahlman in 1982. Emoticons can be used to replace non-verbal communication, especially facial expressions, however, they may not be accurate enough in expressing emotional nuances due to the limitation of the number of keyboard symbols [5]. Thus, emoji are the new generation of emoticons, first developed by Japanese originator Shigetaka Kurita in the late 1990s. The main difference between emoticons and emoji is that emoji are colourful pictures (e.g., 😀 or 🐵) [6]. In those representing facial expressions, a circle is typically used as the boundary, and multiple facial expressions can be expressed within the circle. In other words, emoji are a highly simplified symbolic version of facial expressions, which, compared to emoticons, can express more subtle and complex emotions, and are richer in semantics [7]. By 2015, already 92% of people used emoji in communicating online [1], thus emoji are considered as “the world’s fastest-growing form of communication” [8, p.76]. In addition, they have arguably become a necessary component of online chat. For example, Oleszkiewicz et al. [9]. analysed data from more than 86,000 Facebook users and found that 89.9% of users used at least one emoji in their posts.

Kaye et al. [1] conducted a survey to investigate the reasons why people were inclined to use emoji in online chat. The results demonstrated that sending emoji could promote positive interpersonal relationships. Specifically, emoji could create a relaxed chat atmosphere, promote communication and interaction, and reduce ambiguity in discourse to ensure that the recipient could understand the intended emotional meaning of the message. In addition, Prada et al. [7] argued that the main function of emoji is to express emotion and humour. Furthermore, Thompson et al. [10] found that participants tended to smile more when reading messages that were accompanied by an emoticon compared to the same message without an emoticon.

As well as facilitating online communication, emoji have been widely used in other fields such as marketing—for promotion and to attract attention [11], and to describe consumer emotions [12]. Other domains of application include medicine and public health, where emoji are used to guide people’s behaviour and improve doctor-patient communication [13, 14]. In addition, engineers in computer science use emoji for sentiment analysis to improve unsupervised machine learning [15], and educators apply emoji to improve learning efficiency [16].

However, some studies have shown that people’s interpretation of emoji may still be ambiguous [17, 18], suggesting not only differences in the reasons they use emoji, but also that the comprehension of emoji/emoticons may vary across individuals [19, 20]. Since emoji are such an important part of online communication and are increasingly used in many other fields, it is important to understand the factors that influence their interpretation.

### Facial emotion and emoji

Results from brain imaging studies have suggested that the brain areas activated by recognising real faces have been shown to be similar to those that are activated when viewing more abstract faces such as emoticons [21–23]. Thus, the factors that lead to individual differences in recognition of real faces may also influence people’s ability in understanding emoji depicting facial expressions. Therefore, to determine which kinds of factors may cause individual differences in emoji identification, we first discuss the results from experiments examining real facial emotion recognition.

### Gender

Firstly, emoji comprehension may be influenced by gender. Some previous studies suggest that on average, women demonstrate higher accuracy in emotion recognition than men [24, 25]. One potential explanation is the "primary caretaker hypothesis" [26]. Specifically, accurate and rapid identification of infant emotions, especially facial expressions, is a very important part of infant care, as infant mortality has generally been high throughout human evolution. Regarding specific emotions, some researchers have argued that women are more sensitive to negative facial emotions than men, suggesting a negativity bias [27, 28]. Jones et al. [29] propose that part of the reason is that women have a higher rate of depression from adolescence, and depressed individuals have a stronger negativity bias [30]. However, other researchers have suggested that there are no gender differences in emotion recognition or interpretation [31–33]. Research specifically examining emoji has mainly focused on emoji use rather than interpretation. For example, Chen et al.’s [34] study of 134,419 internet users suggested that males and females differed significantly in emoji usage, and emoji use has been applied in machine learning to predict users’ gender [35]. In the current study, we examine gender differences in emoji interpretation, rather than use. Given that most previous results suggest an accuracy advantage for women in recognising real facial emotions, we expect to observe higher accuracy for women than men in recognising facial expressions depicted by emoji, perhaps more so for negative facial expressions.

### Age

Age may also influence emoji comprehension. A large body of previous research suggests that young adults show higher accuracy in understanding the meaning of emotions than older adults [36, 37]. However, some researchers suggest that older adults are better at recognising emotional facial expressions of disgust than young adults [38, 39]. Hayes et al. [40] suggested that older adults’ recognition of disgust was better than that of young adults due to the stimuli used. Hayes et al. [40] found that older adults were significantly better at recognising disgust from the Pictures of Facial Affect (POFA) [41] than young adults, but were worse than young adults on other image sets. The POFA image set is composed of black and white Ekman faces [41] which may improve the performance of older adults because they were more familiar with black and white images, but the phenomenon only appeared in recognising disgust. This may explain why Calder et al. [38] and Orgeta and Phillips [39]found a tendency for older adults to be better at recognising disgust, since Calder et al. [38] and Orgeta and Phillips [39] showed participants the static photographs from POFA in their experiments. Across studies, the dominant finding suggests that young adults are more accurate than older adults in recognising facial emotions. Therefore, in the present study we predict a negative association between age and accuracy in emoji recognition in general, with the question remaining regarding whether this is also the case for disgust.

### Culture

In addition to gender and age, culture can play an important role in facial emotion recognition. Researchers initially believed that westernised participants had higher accuracy in emotion recognition than participants from other countries [42]. However, Markham and Wang [43] suggested that there might be an in-group advantage whereby participants identified faces from their own group better than faces from other groups. Furthermore, Elfenbein and Ambady [44] conducted a meta-analysis on these cross-cultural emotion recognition experiments. They verified that there was an in-group advantage in emotion recognition, and this advantage would become smaller when one group was exposed to another. Recently, Mandal and Awasthi [45] suggested that there may also be an out-group bias, which means individuals can use decoding rules for out-group facial expressions, and they may be less motivated to recognise the emotions of out-group members. Some underlying mechanisms that have been proposed include language [46], cognitive representations [47], and specific emotional and linguistic experiences in different cultures [48]. Elfenbein and Ambady’s [44] meta-analysis is consistent with this idea—they suggested that people growing up in or being exposed to one culture means that they learn culture-specific elements of emotional behaviour, and this results in an in-group advantage.

In relation to emoji, Kejriwal et al. [49] examined tens of millions of Twitter messages in 30 languages and countries and suggested that usage and interpretation of emoji varied across countries. Guntuku et al. [50] analysed the use of emoji in China and the UK in Twitter and Weibo, and found that usage in China was significantly lower, which indicated that Chinese people are less exposed to emoji. In the present study, we will examine two types of emoji that are frequently used on smartphones: Apple and Android, and one usually seen on computers: Windows. In addition, we will examine the emoji from WeChat, which is widely used in China but rarely used in the UK, to allow us to examine whether any cultural differences are mediated by familiarity and/or platform.

### Aims and hypotheses

Although there is a wealth of literature on individual differences in real facial expression recognition, there are relatively few studies on individual differences in the comprehension of emotional facial expressions depicted by emoji. In the current study, in order to examine whether there are similarities in the classification of facial expressions depicted by emoji, and those of real faces, we investigated individual differences relating to gender, age, and culture in recognising emoji representing six emotions (happy, disgusted, fearful, sad, surprised, and angry, following Ekman & Friesen, [41]) on four different platforms (Apple, Android, Windows, and WeChat). Based on the literature reviewed above, if emoji recognition is similar to emotion recognition in real faces, our hypotheses are: 1) There will be gender differences in emoji classification accuracy, with an accuracy advantage predicted for women, potentially more so for negative emotions, 2) There will be age differences in emoji classification accuracy, with an accuracy advantage for younger participants (although this advantage may not be observed for disgust), 3) There will be cultural differences in emoji recognition accuracy, with an accuracy advantage expected for UK participants, however, this effect may be mediated by familiarity and/or platform.

## Method

### Participants

A power analysis was conducted using the *shiny* package (version 1.7.1) [51]. As both *familiarity* and *platform* would be added into the model as mediators in the analysis of cultural differences, we calculated power using the ‘*Two Parallel Mediators*’ model, where the objective was set to ‘*Set Power*, *Vary N*’. A power level of 0.80, with a minimum sample size of 50, a maximum sample size of 530, and a step size of 10 was selected. In addition, the number of replications was set to 5000, with Monte Carlo Draws per Rep set to 20,000, with a 95% confidence interval, corresponding to an α of 0.05. The correlation coefficients were all set at 0.5, with standard deviations of 1.00. The power analysis demonstrated that a minimum of 130 participants would be required to ensure statistical power of at least 80% for detecting the hypothesised indirect effect of mediator 1, and a minimum of 130 for mediator 2. This adds up to a minimum requirement of 260 participants.

A total of 523 participants were recruited through Prolific, SurveyCircle, and Facebook survey exchange groups. Participants consisted of 253 Chinese and 270 English adults (268 women and 255 men). Ages ranged from 18 to 84 years old (*M* = 36.72, *SD* = 13.39). Participation was voluntary, and participants had the chance to obtain a £20 Amazon voucher via a prize draw. The study was performed in accordance with the ethical standards of the Declaration of Helsinki (University of Nottingham School of Psychology ethics committee reference code: 720). Data were collected between 25^th^ July 2020 and 31^st^ January 2021.

### Materials and design

**Fig 1:**
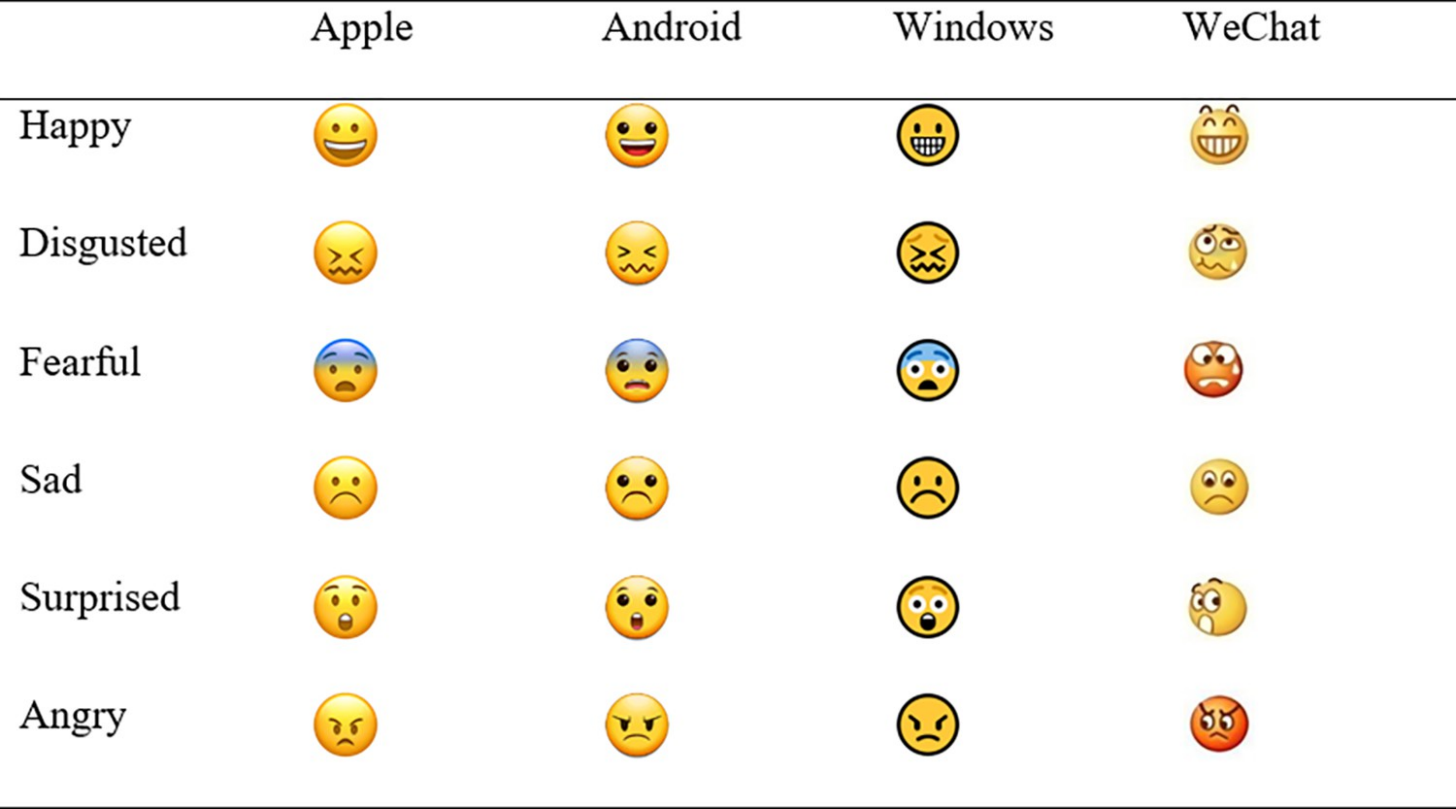
Emoji stimuli for all six emotions across four platforms.

A total of 24 emoji were used in the classification task (see Fig 1). They represented six emotions (happy, disgusted, fearful, sad, surprised, and angry) across four different formats (Apple, Android, Windows, and WeChat). All emoji were 72x72 pixels in size. Android, Apple, and Windows emoji were taken from Unicode Consortium’s Common Locale Data Repository. WeChat emoji were taken from the WeChat official website, as this format was not provided in Unicode. The image size provided in the WeChat website was different from those provided in Unicode (48x48 pixels), so images were magnified to 72x72 pixels to keep the size consistent across platforms. Each emoji was presented to the participants once and in a random order.

### Procedure

There were two versions of the survey: one in English and one in Chinese, and participants were free to choose which one they completed. After giving informed consent, participants were asked for demographic information, such as gender, age, and ethnicity. If participants chose “Chinese” or “English” in the ethnicity column, they could continue to the next step, and if they chose “Other”, they would jump to the end page directly.

Participants then completed the emoji classification task. The 24 emoji were presented one at a time in random order. The task was to indicate which emotion each emoji represented by choosing from six emotional labels. After selecting the emotion label, participants also needed to indicate their familiarity with the emoji. Familiarity was assessed on a five-point scale: "not at all familiar", "slightly familiar", "somewhat familiar", "moderately familiar", "extremely familiar". Participants did not receive feedback about their accuracy. After completing the emoji classification task, participants completed the HEXACO-60 personality scale (as part of a student project). The HEXACO data are not reported here (as they are not relevant to our research questions) and will not be published separately elsewhere. At the end of the experiment, participants were shown a debrief sheet, and given the chance to enter a prize draw to win a £20 Amazon voucher. The study lasted approximately 10–15 minutes, and participants were free to withdraw at any point.

### Data analysis

Data analysis was conducted in *R* (version 4.0.1) [52], where we used causal mediation analysis (*lavaan* package version 0.6–9) [53] to investigate whether there was an effect of gender, age, or culture on emoji recognition, and whether these effects were mediated by familiarity. We separately examined the six types of emoji–happy, surprised, disgusted, fearful, sad, and angry. We created familiarity scores by averaging participants’ familiarity for each emoji type, with a higher score indicating a higher familiarity with that emoji (for reference we have provided the descriptive statistics for the pre-averaged familiarity scores: Not at all familiar = 1, Slightly familiar = 2, Somewhat familiar = 3, Moderately familiar = 4, Extremely familiar = 5).

The first step was to investigate whether there was a direct effect of gender, age, or culture on emoji recognition: for example, *Accuracy ~ Gender*. If there was no direct effect, analysis ended there. If there was a direct effect, the second step was to compare the direct effect and indirect effect (e.g., Accuracy ~ Gender*Familiarity) on emoji recognition, where the effects were calculated with 5,000 bootstrapped samples. For culture analyses, we also included platform as a second mediator, and post-hoc analysis for platform was conducted using the *lsmeans* package (version 2.30–0) [54] utilising a binomial generalised linear model with the significant threshold adjusted using the Bonferroni method. We report detailed results of the analysis, including the following model parameters: estimate (*B*), standard error (*SE*), standardised estimate (β), z-value, *p*-value (*p*), adjusted R-squared (*R*^*2*^), and the 95% confidence intervals. During the pre-processing of the data, missing values were removed, and this process accounted for < 0.001% of the data.

## Results

### Overall emoji recognition accuracy

**Fig 2:**
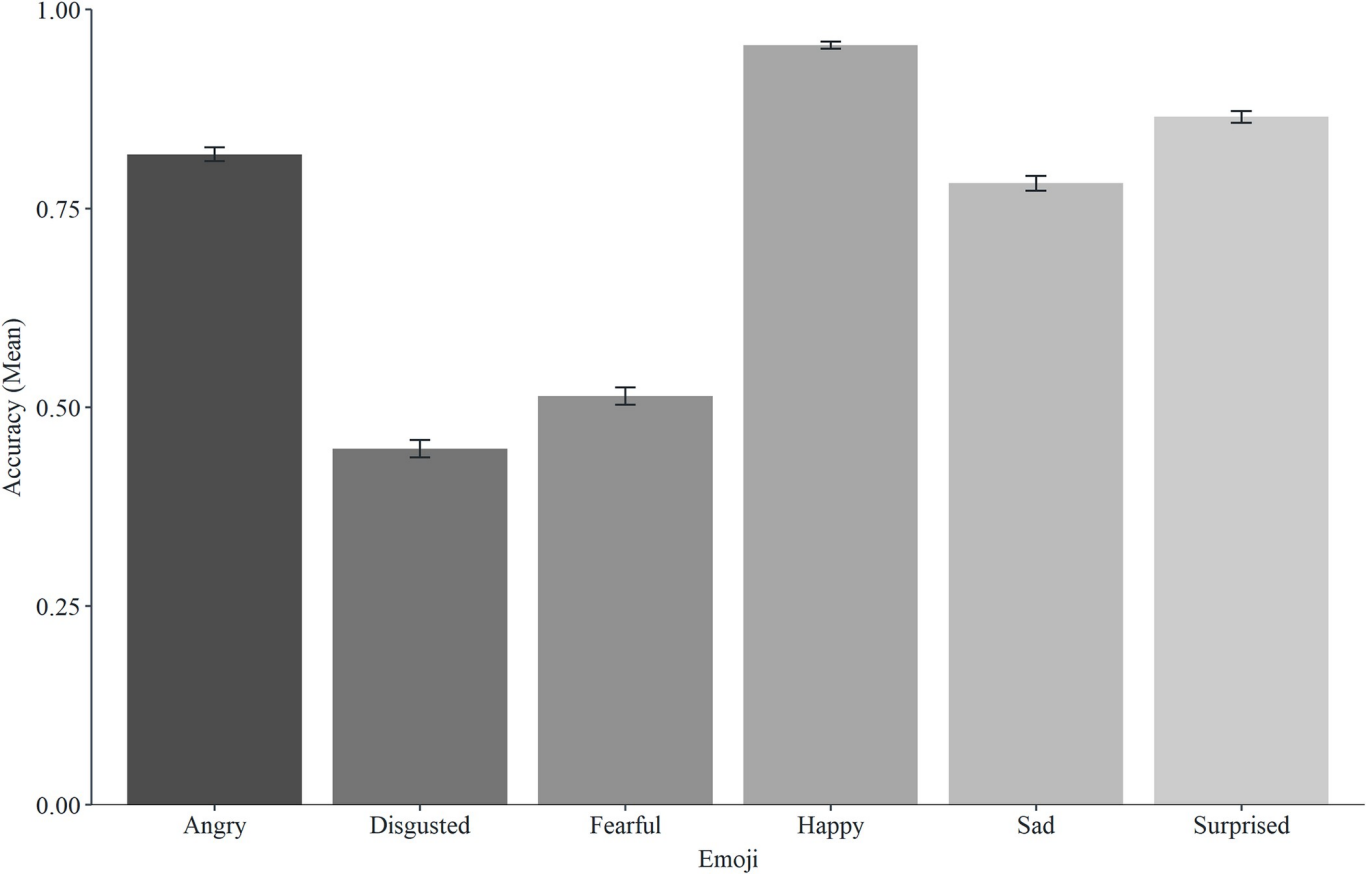
Mean accuracy scores for the six emoji (error bars represent SEM).

**Fig 3:**
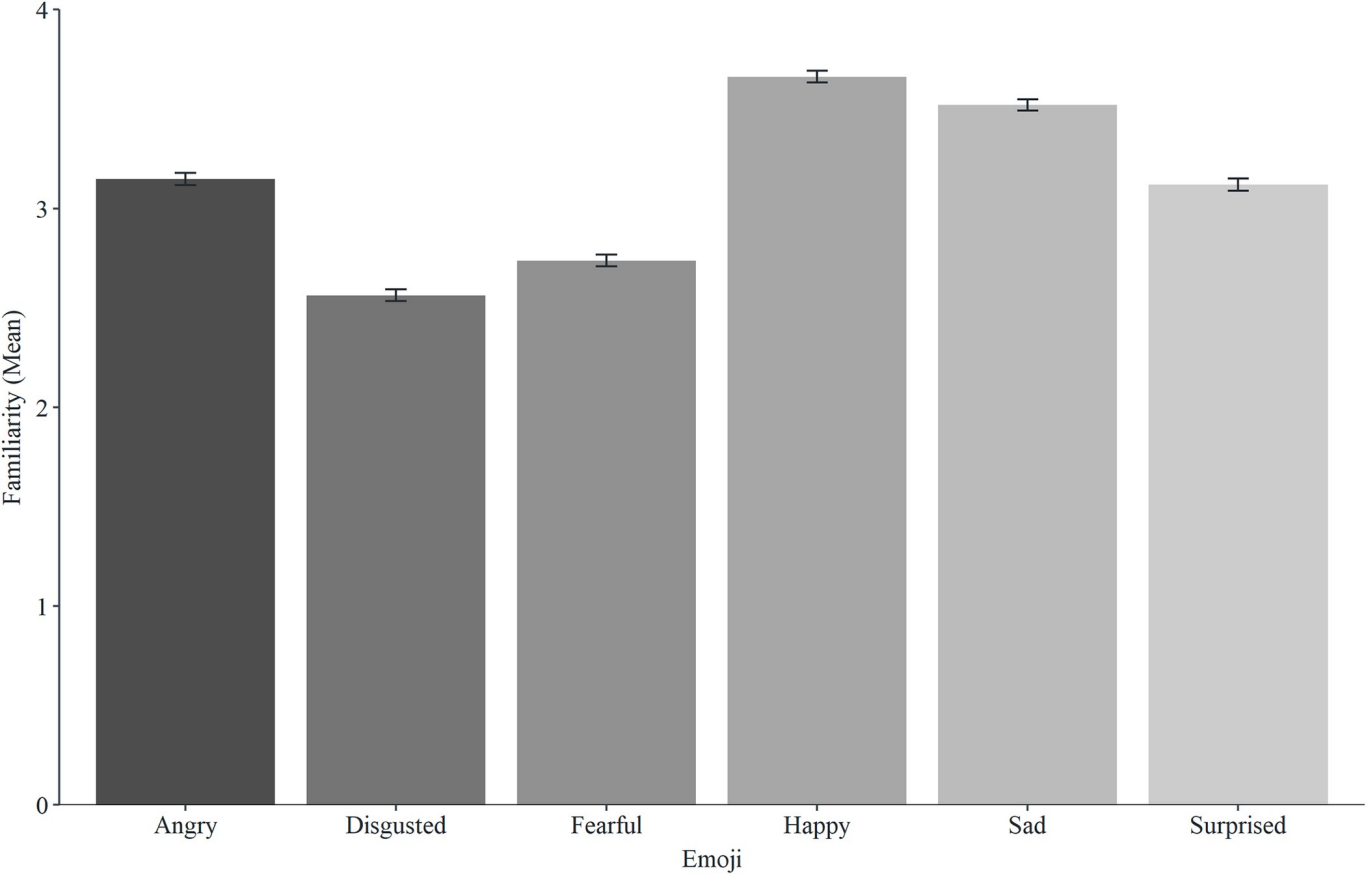
Mean familiarity scores for the six emoji (error bars represent SEM).

See Figs 2 and 3 for accuracy and familiarity descriptive statistics for all emoji. A one-way MANOVA was conducted to determine whether the type of emoji had an effect on accuracy and familiarity. Using Pillai’s trace, there was a significant difference in accuracy and familiarity based on the type of emoji, *V* = 0.89, *F*(10, 25074) = 307, *p* < .001, partial ƞ*2* = .11. Separate univariate ANOVAs on the variables revealed significant effects of type of emoji on accuracy, *F*(5, 12537) = 527.79, *p* < .001, and familiarity *F*(5, 12537) = 207.96, *p* < .001. To directly address our predictions, we now present separate analyses focusing on each individual difference factor in turn (gender, age, and culture).

### Gender effects

**Fig 4:**
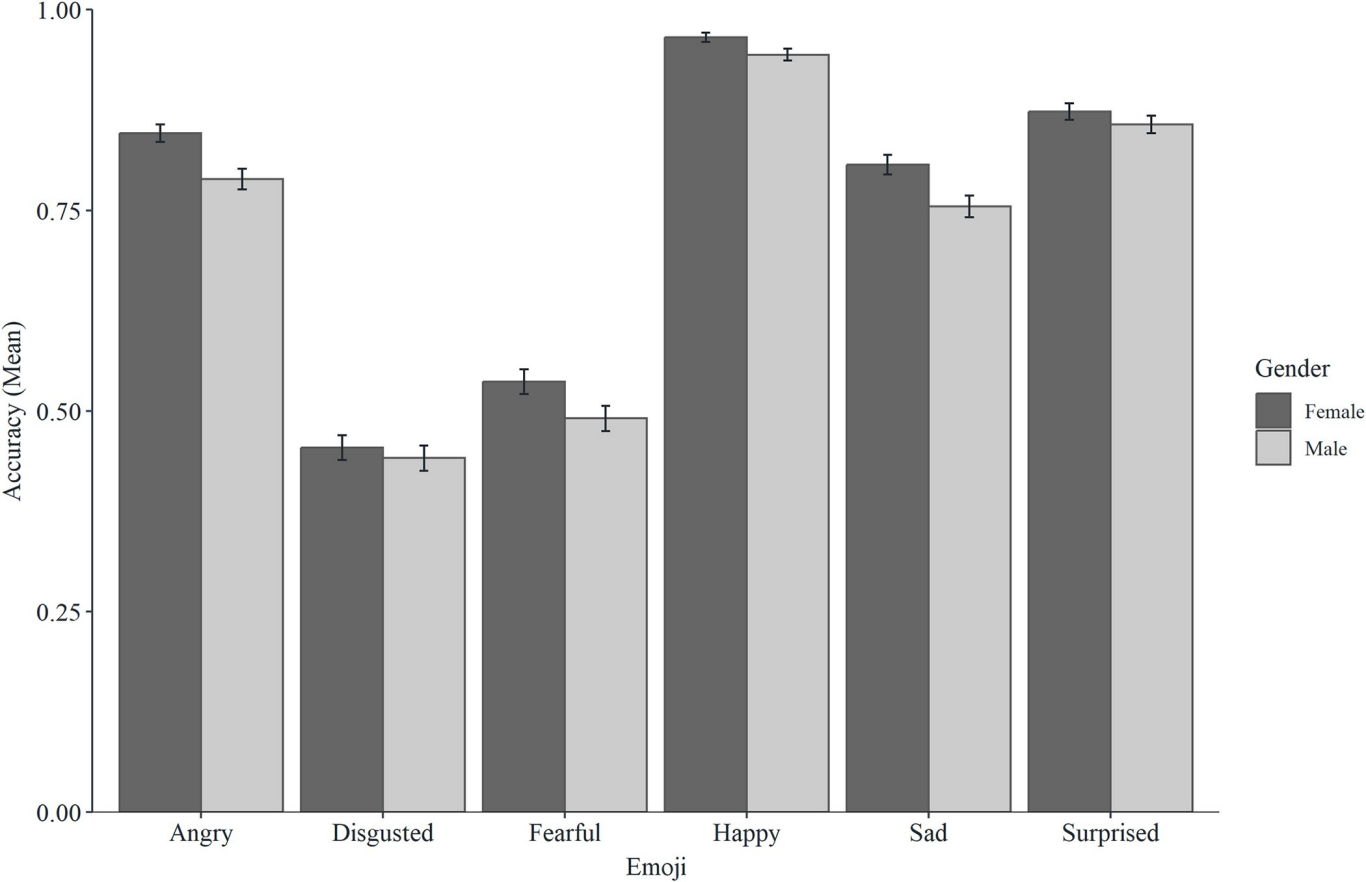
Mean accuracy scores for the six emoji across genders (error bars represent SEM).

**Fig 5:**
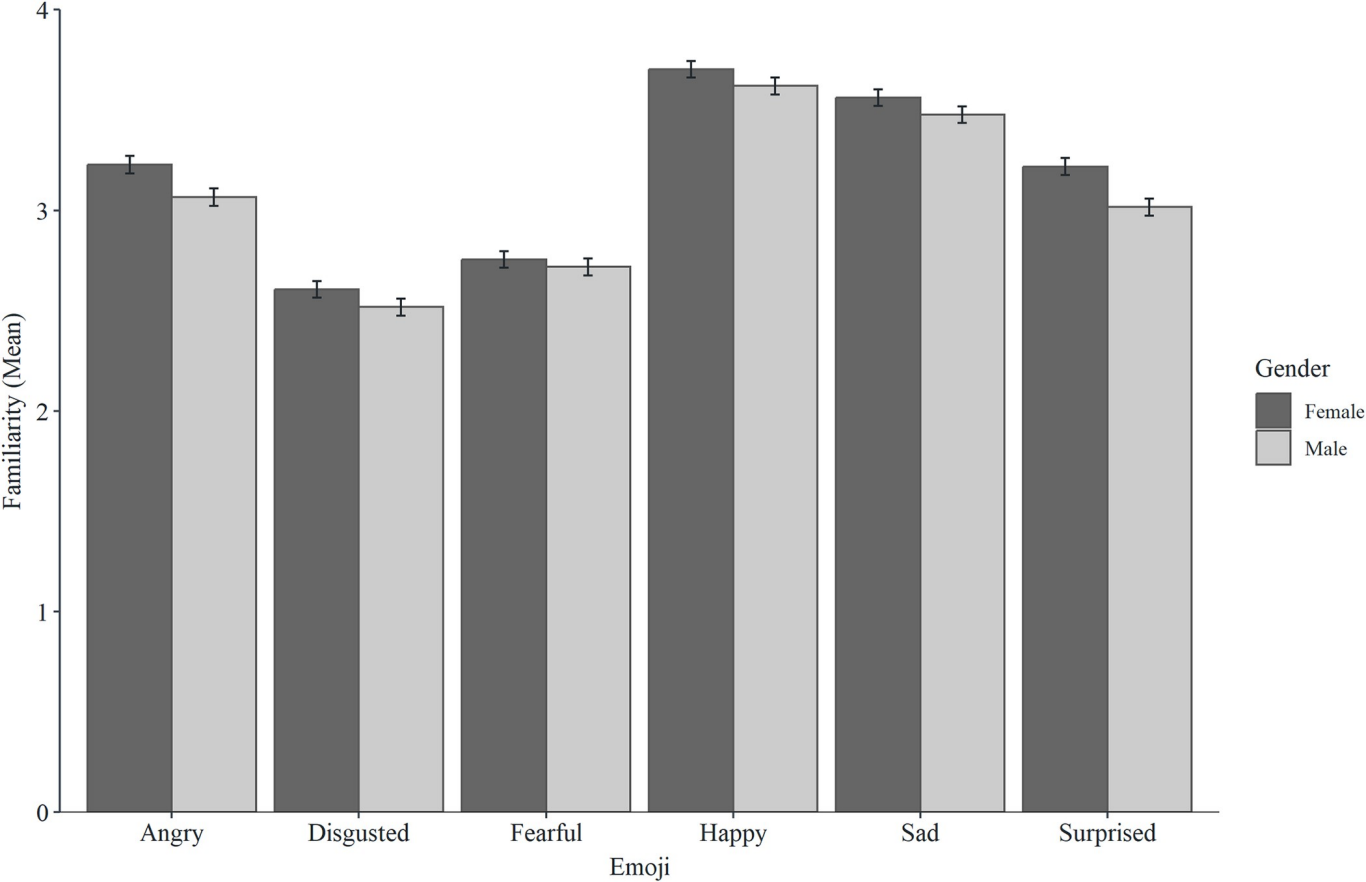
Mean familiarity scores for the six emoji across genders (error bars represent SEM).

There were significant direct effects of gender for happy, fearful, sad, and angry emoji, showing higher classification accuracy for women than men. Mediation analyses showed that accuracy effects were not mediated by familiarity for happy, fearful, or sad emoji, but were partially (but not fully) mediated by familiarity for angry emoji. There were no direct effects of gender for surprised or disgusted emoji (see Figs 4 and 5 for descriptive statistics and Table 1 for mediation analysis).

**Table 1:** Gender: Models, parameters, and Confidence Intervals (CI).

Emoji	Variable	Model	*B*	β	95% CI (2.5%, 97.5%)		*SE*	*z*	*p*
Happy	Direct	*Accuracy ~ Gender (c)*	-0.020	-0.047	-0.038	-0.002	0.009	-2.184	0.03
		*Familiarity ~ Gender (a)*	-0.090	-0.048	-0.171	-0.005	0.042	-2.145	0.03
		*Accuracy ~ Familiarity (b)*	0.021	0.094	0.010	.031	0.005	3.855	< 0.001
	Indirect	Accuracy ~ Gender*Familiarity (a*b)	-0.002	-0.004	-0.004	-0.000	0.001	-1.869	0.06
	*R* ^*2*^	*Accuracy*	0.012						
		*Familiarity*	0.002						
Surprised	Direct	*Accuracy ~ Gender (c)*	-0.016	-0.024	-0.046	0.013	0.015	-1.087	0.28
	*R* ^*2*^	*Accuracy*	0.001						
Disgusted	Direct	*Accuracy ~ Gender (c)*	-0.013	-0.013	-0.056	0.029	0.022	-0.607	0.54
	*R* ^*2*^	*Accuracy*	0.000						
Fearful	Direct	*Accuracy ~ Gender (c)*	-0.045	-0.045	-0.089	-0.001	0.022	-2.023	0.04
		*Familiarity ~ Gender (a)*	-0.045	-0.022	-0.135	0.045	0.046	-0.972	0.33
		*Accuracy ~ Familiarity (b)*	0.018	0.038	-0.002	0.040	0.011	1.702	0.09
	Indirect	Accuracy ~ Gender*Familiarity (a*b)	-0.001	-0.001	-0.004	0.001	0.001	-0.735	0.46
	*R* ^*2*^	*Accuracy*	0.004						
		*Familiarity*	0.000						
Sad	Direct	*Accuracy ~ Gender (c)*	-0.044	-0.054	-0.079	-0.009	0.018	-2.503	0.01
		*Familiarity ~ Gender (a)*	-0.085	-0.041	-0.173	0.003	0.045	-1.881	0.06
		*Accuracy ~ Familiarity (b)*	0.091	0.228	0.075	0.107	0.008	11.005	< 0.001
	Indirect	Accuracy ~ Gender*Familiarity (a*b)	-0.008	-0.009	-0.016	0.000	0.004	-1.835	0.07
	*R* ^*2*^	*Accuracy*	0.056						
		*Familiarity*	0.002						
Angry	Direct	*Accuracy ~ Gender (c)*	-0.050	-0.065	-0.083	-0.017	0.017	-2.974	0.003
		*Familiarity ~ Gender (a)*	-0.164	-0.078	-0.255	-0.071	0.047	-3.520	< 0.001
		*Accuracy ~ Familiarity (b)*	0.043	0.117	0.026	0.058	0.008	5.323	< 0.001
	Indirect	Accuracy ~ Gender*Familiarity (a*b)	-0.007	-0.009	-0.012	-0.003	0.002	-2.916	0.004
	*R* ^*2*^	*Accuracy*	0.019						
		*Familiarity*	0.006						

### Age effects

There were significant direct effects of age for surprised, fearful, sad, and angry emoji, such that the older the participant, the less accurate they were. Effects were not mediated by familiarity for surprised or fearful emoji, and were partially (but not fully) mediated by familiarity for sad and angry emoji. The direct effect of age for disgusted emoji was removed by mediation analysis, and there were no direct effects of age for happy emoji (see Table 2 for mediation analysis).

**Table 2:** Age: Models, parameters, and Confidence Intervals (CI).

Emoji	Variable	Model	*B*	β	95% CI (2.5%, 97.5%)		*SE*	*z*	*p*
Happy	Direct	*Accuracy ~ Age (c)*	-0.000	-0.017	-0.001	0.000	0.000	-0.789	0.43
	*R* ^*2*^	*Accuracy*	0.000						
Surprised	Direct	*Accuracy ~ Age (c)*	-0.005	-0.186	-0.006	-0.004	0.001	-7.466	< 0.001
	Indirect	*Familiarity ~ Age (a)*	-0.030	-0.398	-0.033	-0.027	0.002	-19.955	< 0.001
		*Accuracy ~ Familiarity (b)*	0.003	0.010	-0.013	0.019	0.008	0.417	0.68
		Accuracy ~ Age*Familiarity (a*b)	-0.000	-0.004	-0.001	0.000	0.000	-0.416	0.68
	*R* ^*2*^	*Accuracy*	0.036						
		*Familiarity*	0.158						
Disgusted	Direct	*Accuracy ~ Age (c)*	-0.001	-0.036	-0.003	0.000	0.001	-1.575	0.12
	Indirect	*Familiarity ~ Age (a)*	-0.029	-0.358	-0.032	-0.026	0.002	-18.097	< 0.001
		*Accuracy ~ Familiarity (b)*	0.009	0.019	-0.013	0.031	0.011		
		Accuracy ~ Age*Familiarity (a*b)	-0.000	-0.007	-0.001	0.000	0.000	0.799	0.42
	*R* ^*2*^	*Accuracy*	0.002					-0.795	0.43
		*Familiarity*	0.128						
Fearful	Direct	*Accuracy ~ Age (c)*	-0.003	-0.081	-0.005	-0.001	0.001	-3.346	0.001
		*Familiarity ~ Age (a)*	-0.028	-0.366	-0.031	-0.025	0.002	-18.314	< 0.001
		*Accuracy ~ Familiarity (b)*	0.004	0.009	-0.018	0.027	0.011	0.384	0.70
	Indirect	Accuracy ~ Age*Familiarity (a*b)	-0.000	-0.003	-0.001	0.001	0.000	-0.383	0.70
	*R* ^*2*^	*Accuracy*	0.007						
		*Familiarity*	0.134						
Sad	Direct	*Accuracy ~ Age (c)*	-0.005	-0.150	-0.006	-0.003	0.001	-6.565	< 0.001
		*Familiarity ~ Age (a)*	-0.022	-0.280	-0.025	-0.018	0.002	-13.403	< 0.001
		*Accuracy ~ Familiarity (b)*	0.075	0.188	0.058	0.092	0.009	8.710	< 0.001
	Indirect	Accuracy ~ Age*Familiarity (a*b)	-0.002	-0.053	-0.002	-0.001	0.000	-7.342	< 0.001
	*R* ^*2*^	*Accuracy*	0.074						
		*Familiarity*	0.079						
Angry	Direct	*Accuracy ~ Age (c)*	-0.006	-0.203	-0.007	-0.004	0.001	-8.217	< 0.001
		*Familiarity ~ Age (a)*	-0.029	-0.373	-0.033	-0.026	0.002	-18.573	< 0.001
		*Accuracy ~ Familiarity (b)*	0.017	0.046	-0.000	0.033	0.008	1.993	0.05
	Indirect	Accuracy ~ Age*Familiarity (a*b)	-0.000	-0.017	-0.001	0.000	0.000	-1.971	0.05
	*R* ^*2*^	*Accuracy*	0.050						
		*Familiarity*	0.139						

### Culture effects

**Fig 6:**
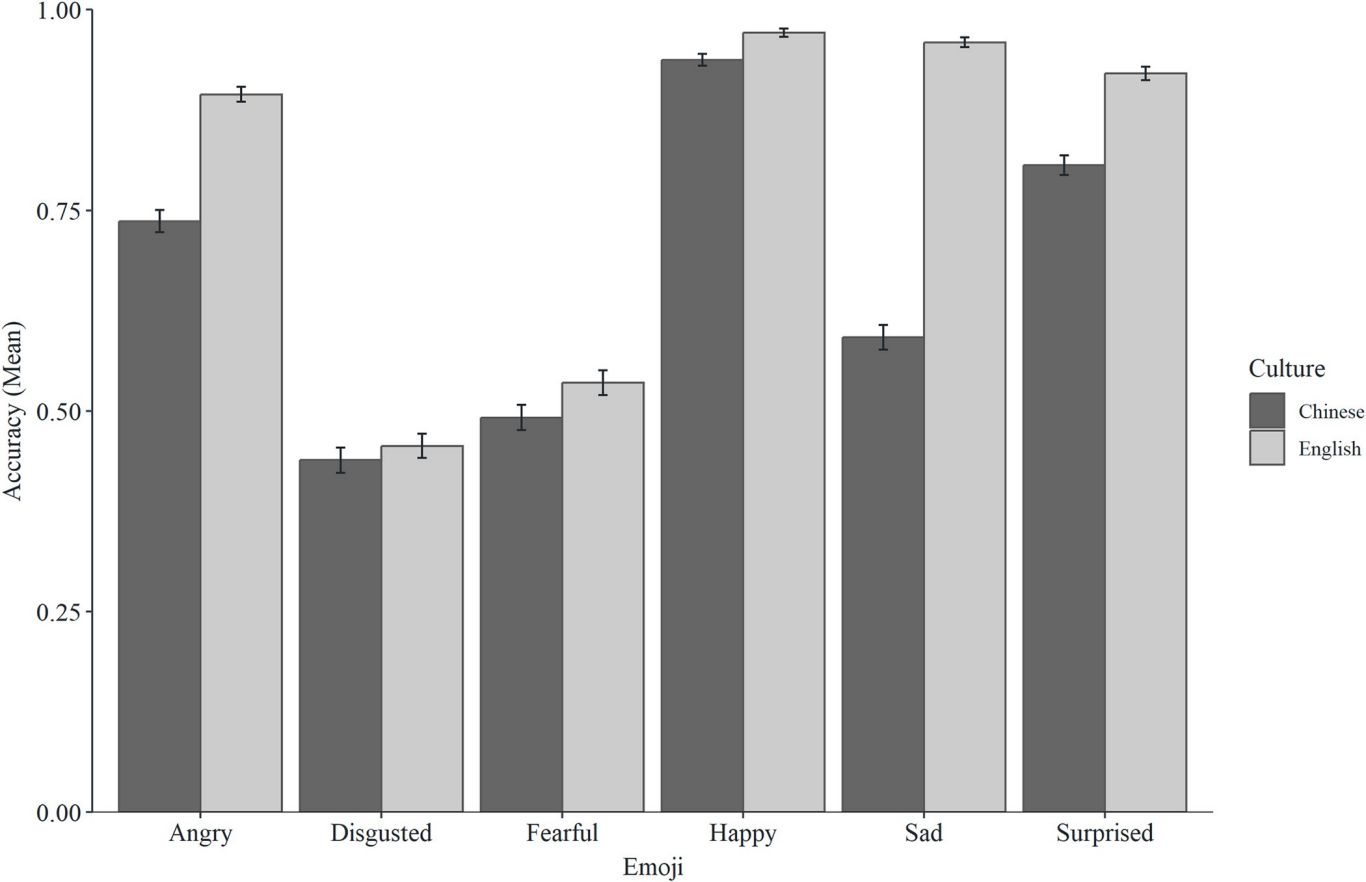
Mean accuracy scores for the six emoji across cultures (error bars represent SEM).

**Fig 7:**
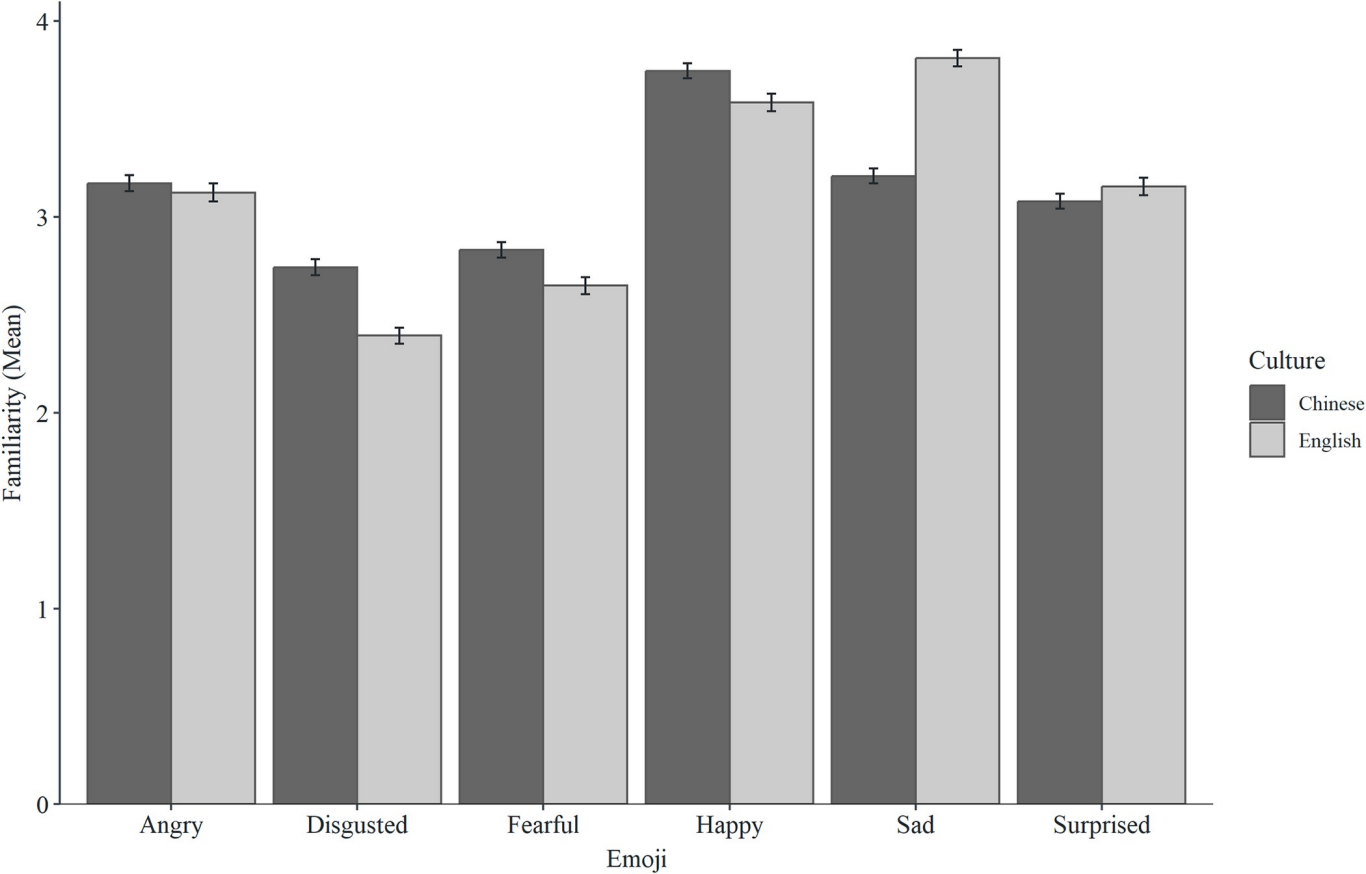
Mean familiarity scores for the six emoji across cultures (error bars represent SEM).

There were significant direct effects of culture for happy, sad, surprised, fearful, and angry emoji, such that participants from the UK had higher accuracy scores than Chinese participants. These effects were not mediated by familiarity for surprise, fear, or angry, and were partially (but not fully) mediated by familiarity for happy and sad emoji. None of the direct effects were mediated by platform. There was no direct effect of culture for disgusted emoji (see Figs 6 and 7 and Table 3 for descriptive statistics, and see Table 4 for mediation analysis).

**Table 3:** Descriptive statistics: Culture and platform–accuracy.

Emoji	Variable(s)	Mean	Standard Error of the Mean (SEM)
Happy	English: Android	0.99	0.007
	English: Apple	0.99	0.006
	English: WeChat	0.96	0.01
	English: Windows	0.95	0.01
	Chinese: Android	0.95	0.01
	Chinese: Apple	0.95	0.01
	Chinese: WeChat	0.94	0.01
	Chinese: Windows	0.90	0.02
Surprised	English: Android	0.98	0.009
	English: Apple	0.98	0.01
	English: WeChat	0.79	0.03
	English: Windows	0.95	0.01
	Chinese: Android	0.80	0.03
	Chinese: Apple	0.84	0.02
	Chinese: WeChat	0.84	0.02
	Chinese: Windows	0.74	0.03
Disgusted	English: Android	0.53	0.53
	English: Apple	0.46	0.46
	English: WeChat	0.50	0.50
	English: Windows	0.34	0.34
	Chinese: Android	0.42	0.03
	Chinese: Apple	0.39	0.03
	Chinese: WeChat	0.63	0.03
	Chinese: Windows	0.32	0.03
Fearful	English: Android	0.64	0.03
	English: Apple	0.46	0.03
	English: WeChat	0.54	0.03
	English: Windows	0.50	0.03
	Chinese: Android	0.49	0.03
	Chinese: Apple	0.43	0.03
	Chinese: WeChat	0.30	0.03
	Chinese: Windows	0.45	0.03
Sad	English: Android	0.96	0.01
	English: Apple	0.99	0.006
	English: WeChat	0.91	0.02
	English: Windows	0.98	0.009
	Chinese: Android	0.56	0.03
	Chinese: Apple	0.61	0.03
	Chinese: WeChat	0.57	0.03
	Chinese: Windows	0.62	0.03
Angry	English: Android	0.94	0.01
	English: Apple	0.95	0.01
	English: WeChat	0.74	0.03
	English: Windows	0.95	0.01
	Chinese: Android	0.72	0.03
	Chinese: Apple	0.73	0.03
	Chinese: WeChat	0.82	0.02
	Chinese: Windows	0.67	0.03

**Table 4:** Culture: Models, parameters, and Confidence Intervals (CI).

Emoji	Variable	Model	*B*	β	95% CI (2.5%, 97.5%)		*SE*	*z*	*p*
Happy	Direct	*Accuracy ~ Culture (c)*	0.037	0.090	0.019	0.055	0.009	4.041	< 0.001
		*Familiarity ~ Culture (a1)*	-0.156	-0.082	-0.239	-0.075	0.041	-3.769	< 0.001
		*Platform ~ Culture (a2)*	-0.000	-0.000	-0.098	0.098	0.050	-0.010	0.99
		*Accuracy ~ Familiarity (b1)*	0.023	0.104	0.012	0.034	0.005	4.217	< 0.001
		*Accuracy ~ Platform (b2)*	-0.015	-0.078	-0.023	-0.006	0.004	-3.441	0.001
	Indirect	Accuracy ~ Culture*Familiarity (a1*b1)	-0.004	-0.009	-0.006	-0.001	0.001	-2.753	0.006
		Accuracy ~ Culture*Platform (a2*b2)	0.000	0.000	-0.002	0.002	0.001	0.010	0.99
		*Familiarity ~~ Platform*	-0.000	-0.000	-0.045	0.046	0.023	-0.002	1.00
	Covariance	*Accuracy*	0.024						
	*R* ^*2*^	*Familiarity*	0.007						
		*Platform*	0.000						
Surprised	Direct	*Accuracy ~ Culture (c)*	0.112	0.164	0.084	0.141	0.015	7.641	< 0.001
		*Familiarity ~ Culture (a1)*	0.076	0.038	-0.010	0.161	0.044	1.741	0.08
		*Platform ~ Culture (a2)*	-0.000	-0.000	-0.095	0.094	0.049	-0.000	1.00
		*Accuracy ~ Familiarity (b1)*	0.026	0.078	0.012	0.041	0.007	3.618	< 0.001
		*Accuracy ~ Platform (b2)*	-0.023	-0.076	-0.036	-0.011	0.006	-3.611	< 0.001
	Indirect	Accuracy ~ Culture*Familiarity (a1*b1)	0.002	0.003	-0.000	0.005	0.001	1.533	0.13
		Accuracy ~ Culture*Platform (a2*b2)	0.000	0.000	-0.002	0.002	0.001	0.000	1.00
		*Familiarity ~~ Platform*	-0.000	-0.000	-0.049	0.048	0.025	-0.000	1.00
	Covariance	*Accuracy*	0.040						
	*R* ^*2*^	*Familiarity*	0.001						
		*Platform*	0.000						
Disgusted	Direct	*Accuracy ~ Culture (c)*	0.018	0.018	-0.024	0.060	0.021	0.831	0.41
	*R* ^*2*^	*Accuracy*	0.000						
Fearful	Direct	*Accuracy ~ Culture (c)*	0.047	0.047	0.005	0.090	0.022	2.144	0.03
		*Familiarity ~ Culture (a1)*	-0.175	-0.085	-0.260	-0.085	0.045	-3.884	< 0.001
		*Platform ~ Culture (a2)*	-0.003	-0.002	-0.097	0.094	0.049	-0.071	0.94
		*Accuracy ~ Familiarity (b1)*	0.021	0.043	-0.000	0.041	0.011	1.939	0.05
		*Accuracy ~ Platform (b2)*	-0.015	-0.034	-0.034	0.005	0.010	-1.569	0.12
	Indirect	Accuracy ~ Culture*Familiarity (a1*b1)	-0.004	-0.004	-0.008	0.000	0.002	-1.689	0.09
		Accuracy ~ Culture*Platform (a2*b2)	0.000	0.000	-0.002	0.002	0.001	0.060	0.95
		*Familiarity ~~ Platform*	-0.000	-0.000	-0.051	0.050	0.025	-0.010	0.99
	Covariance	*Accuracy*	0.005						
	*R* ^*2*^	*Familiarity*	0.007						
		*Platform*	0.000						
Sad	Direct	*Accuracy ~ Culture (c)*	0.341	0.412	0.307	0.375	0.017	19.767	< 0.001
		*Familiarity ~ Culture (a1)*	0.602	0.290	0.515	0.686	0.043	14.077	< 0.001
		*Platform ~ Culture (a2)*	0.000	0.000	-0.098	0.095	0.050	0.000	1.00
		*Accuracy ~ Familiarity (b1)*	0.044	0.110	0.028	0.060	0.008	5.468	< 0.001
		*Accuracy ~ Platform (b2)*	0.007	0.019	-0.007	0.021	0.007	0.999	0.32
	Indirect	Accuracy ~ Culture*Familiarity (a1*b1)	0.026	0.032	0.016	0.037	0.005	5.084	< 0.001
		Accuracy ~ Culture*Platform (a2*b2)	0.000	0.000	-0.001	0.001	0.001	0.000	1.00
		*Familiarity ~~ Platform*	-0.000	-0.000	-0.048	0.046	0.024	-0.000	1.00
	Covariance	*Accuracy*	0.209						
	*R* ^*2*^	*Familiarity*	0.084						
		*Platform*	0.000						
Angry	Direct	*Accuracy ~ Culture (c)*	0.160	0.207	0.128	0.193	0.016	9.698	< 0.001
		*Familiarity ~ Culture (a1)*	-0.041	-0.020	-0.132	0.047	0.046	-0.905	0.37
		*Platform ~ Culture (a2)*	0.001	0.001	-0.091	0.099	0.049	0.031	0.98
		*Accuracy ~ Familiarity (b1)*	0.046	0.126	0.031	0.061	0.008	5.965	< 0.001
		*Accuracy ~ Platform (b2)*	-0.014	-0.040	-0.027	-0.000	0.007	-12.003	0.05
	Indirect	Accuracy ~ Culture*Familiarity (a1*b1)	-0.002	-0.002	-0.006	0.002	0.002	-0.886	0.38
		Accuracy ~ Culture*Platform (a2*b2)	-0.000	-0.000	-0.002	0.002	0.001	-0.027	0.98
		*Familiarity ~~ Platform*	0.001	0.001	-0.049	0.051	0.026	0.035	0.97
	Covariance	*Accuracy*	0.059						
	*R* ^*2*^	*Familiarity*	0.000						
		*Platform*	0.000						

## Discussion

The aim of the present study was to investigate individual differences relating to gender, age, and culture, in classifying emoji representing facial emotional expressions. Overall, the results suggested that all of the factors under investigation (gender, age, and culture) had a significant impact on how emoji were classified.

### Gender effects

The findings relating to gender showed some classification accuracy advantages for women, which would support previous research on facial emotion recognition [24, 25], However, it does not necessarily support the suggestion that this is due to women displaying a negativity bias [27, 28], as women also showed higher accuracy for ‘happy’ emoji in the current study.

Notably, women were not more accurate in recognising all emoji types; there were no gender differences for surprised or disgusted emoji. The identification of surprise necessitates processing of both the upper (the eyes) and lower parts (nose and mouth) of the face [55–57]. According to the findings of Abbruzzese et al. [58] and Sullivan et al. [57], females were more likely to look at the eyes and males looked more to the mouth, which may result in similarly high accuracy in identifying surprised expressions for both genders.

Interestingly, recognition of disgust was not influenced by gender, age, or culture. Previous facial expression research [59, 60] and emoji research [61] suggested that disgust and fear are least-well recognised; a finding mirrored in the present results. According to Lambrecht et al. [62], there was no difference between females and males in recognising disgust due to disgust rarely appearing in our daily life [63].

### Age effects

The results showed an overall accuracy advantage for younger participants, apart from happy and disgusted emoji. This finding partly converges with previous findings that young adults were more accurate than older adults in recognising emotions [36, 37]. It also accords with previous findings suggesting that the recognition of disgust is preserved during aging [38, 39], and further suggests that this finding may not entirely be due to the black and white stimuli used in previous studies. It may be that compared with younger adults, older adults were more likely to focus on the bottom half of the face (nose and mouth), leading to better recognition of disgusted and happy emotions, which could increase the accuracy of older adults and decrease the difference between younger and older individuals [55, 57]. The lack of an age-related decline for recognising happy emoji could also be due to the purported ‘positivity bias’ [64] observed in older adults, facilitating recognition in this case.

Although the current findings could be interpreted as suggesting that older adults may benefit less from the paralinguistic cues provided by emoji than young adults (at least for some emotions), it is important to note that some recent research has suggested that older adults may benefit from the use of emoji to clarify the meaning of more complex and ambiguous messages, such as those intended to be interpreted sarcastically [65]. Thus, further research is needed to investigate age-related differences in the interpretation of emoji both in isolation and in conjunction with written messages.

### Culture effects

Results showed a general accuracy advantage for UK compared to Chinese participants, in that UK participants were more accurate in identifying all emotions except disgust. These effects were partially mediated by familiarity for happy and sad emoji only, suggesting that the effect cannot be fully explained by UK participants being more familiar with the emoji that were used. The lack of mediation by platform would also support this suggestion. However, even though Chinese people use WeChat much more frequently than people in the UK, they may nevertheless not be regular users of WeChat emoji. A recent report [66] showed that the type of emoji they used were very limited.

Additionally, Chinese internet users may use emoji for different communication purposes—for example, it has been suggested that they seldom use the happy emoji to express happiness, instead, they use it for negative meanings such as sarcasm [67]. Similarly, Guntuku et al. [50] analysed the emoji posted on Twitter and Weibo in four countries and demonstrated that in some areas, Eastern people tended to refer to the same thing with entirely different emoji compared to Western people, and this is also supported by Kejriwal et al. [49]. Thus, Chinese participants may show lower ‘accuracy’ because they tend to use these emoji in a different way. Finally, the differences observed between UK and Chinese participants may also be due to distinctions between the six ‘universal’ emotions that were used in the current study (following Ekman & Friesen, 41) being less clear cut for Eastern than Western participants [68]. This suggests the need for future research focusing on a more nuanced examination of cultural differences in emoji identification and use across different contexts.

### Limitations and future directions

There are some limitations to the current study which need to be considered. As discussed above, the current experiment examined the interpretation of emoji in isolation, to allow us to assess whether similar factors influence the recognition of facial expressions depicted by emoji as those that influence real facial expressions. Like real facial expressions, emoji are more frequently interpreted in context, which may lead to more nuanced effects. In addition, the current study focused on emoji representing the six ‘universal’ emotions, and interestingly, different effects were observed for different emotions, suggesting that any accuracy advantages observed may be related to specific emoji. Thus, future studies should examine individual differences in the interpretation of a wider selection of frequently used emoji both in and out of context. Interestingly, recent research investigating emotions that are evoked by more complex situations (using video-based stimuli) has developed cutting-edge techniques to examine individual differences in emotion profiles [69]. Such an approach could be adopted in future research on emoji, particularly when considering responses to emoji conveying more complex, context-dependent information than those investigated in the current study.

Finally, in our study, we did not ask participants for demographic information beyond gender, age, and culture. In future, it would be interesting to consider a more detailed profile of the participants, including further individual differences which might be relevant to facial emotion recognition ability. Indeed, there is emerging evidence that autistic traits, alexithymia, and attachment styles may interact to influence emoji classification accuracy [e.g., 70], but it is as yet unknown how these individual differences factors may interact with the factors investigated in the current study. In relation to this, a further interesting avenue for research would be to examine whether incorporating more visual cues can facilitate online communication in these diverse populations.

## Conclusions

In conclusion, the results from the current study revealed a range of individual differences in how participants classified emoji representing facial emotional expressions. Although a number of findings were in line with those from previous studies on ‘real’ facial emotion recognition, suggesting that similar individual differences may come into play across the two domains, the whole story is likely to be more complex. For instance, our findings in relation to age and culture highlight the importance of context in emoji use, for example, the possibility that participants in China may commonly use the ‘smile’ emoji for different purposes than to signify happiness, which means some ‘universal’ facial emotions may not be ‘universal’ when they transfer to emoji. The current results have important implications when considering emoji use in online communication, for example, with conversation partners from different cultures or of different ages. Given the broad and expanding use of emoji in other domains, the findings of individual differences in their interpretation also has more wide-reaching implications—for instance, in improving classification accuracy in sentiment analysis [71, 72], and regarding digital advertising within marketing, multinational corporations may need to apply different emoji for marketing purposes in different nations.
